# From Skin to Sinus: A Rare Case of Cerebral Venous Sinus Thrombosis Caused by Occipital Subcutaneous Abscess

**DOI:** 10.7759/cureus.84594

**Published:** 2025-05-22

**Authors:** Koh Ishida, Tatsuya Tanaka, Aiko Saku, Takamichi Nakajima, Hirotoshi Kawashima, Akira Matsuno

**Affiliations:** 1 Neurosurgery, International University of Health and Welfare Narita Hospital, Narita, JPN; 2 Allergy and Rheumatology, International University of Health and Welfare Narita Hospital, Narita, JPN; 3 Emergency, International University of Health and Welfare Narita Hospital, Narita, JPN

**Keywords:** anticoagulation therapy, antimicrobial therapy, cerebral venous sinus thrombosis, emissary veins, headache, intracranial infection, methicillin-sensitive staphylococcus aureus, septic thrombosis, subcutaneous abscess, surgical drainage

## Abstract

Infectious cerebral venous sinus thrombosis (CVST) typically arises from contiguous infections such as sinusitis or otitis media; however, CVST secondary to a subcutaneous abscess is exceedingly rare.

We report the case of a 68-year-old woman who presented with a fever, headache, and a painful occipital scalp mass. Initial imaging identified a subcutaneous abscess in the occipital region, and subsequent magnetic resonance imaging (MRI) revealed thrombosis involving the superior sagittal, transverse, and sigmoid sinuses. Methicillin-sensitive Staphylococcus aureus (MSSA) was isolated from both blood and abscess cultures. Anticoagulation and intravenous antibiotic therapy were promptly initiated. Diplopia developed on day 10 of illness, was closely monitored through serial neurological examinations, and gradually resolved over the ensuing months, with complete resolution noted at the six-month follow-up. Follow-up imaging at 24 months demonstrated partial recanalization of the affected venous sinuses.

This case underscores a rare but critical progression from a localized scalp infection to CVST. Clinicians should maintain a high index of suspicion in patients presenting with occipital scalp infections accompanied by neurological symptoms. To our knowledge, this is one of the few reported cases of CVST arising from a posterior scalp abscess, expanding the spectrum of infectious sources and guiding management strategies in similar presentations.

## Introduction

Subcutaneous abscesses are localized bacterial infections of the skin or subcutaneous tissue that typically resolve with antibiotic therapy, incision, and drainage [1]. However, infections complicated by highly pathogenic microorganisms, compromised host immunity, and delayed initiation of treatment can lead to the spread of local infections to surrounding tissues or through the bloodstream, resulting in severe complications.

Cerebral venous sinus thrombosis (CVST) is a condition in which a thrombus forms within the venous sinuses responsible for venous drainage of the brain. This condition causes a range of symptoms, including headache, nausea, vomiting, seizures, visual disturbances, altered consciousness, and focal neurological deficits [2-5]. CVST associated with head and neck infections, such as sinusitis, otitis media, and mastoiditis, is relatively well-documented [2-6]. Cases of CVST accompanied by subcutaneous abscesses arising from otolaryngologic infections have also been reported [7,8]. However, CVST secondary to subcutaneous abscesses that are not associated with otolaryngologic infections remains exceedingly rare [9].

Here, we report a case of CVST extending from the superior sagittal sinus to the right sigmoid sinus, which occurred following Methicillin-Susceptible Staphylococcus aureus (MSSA) bacteremia originating from an occipital subcutaneous abscess.

## Case presentation

A 68-year-old female with no significant past medical history, occasional alcohol intake, and no history of smoking presented with an occipital headache that had gradually worsened over several days, accompanied by a persistent fever exceeding 38°C. On day 5 of symptom onset, she visited a local clinic and was prescribed loxoprofen; however, her symptoms remained unchanged. On day 6, based on a suspected diagnosis of polymyalgia rheumatica, prednisolone (15 mg/day) was initiated. On day 9, she presented to the emergency department of our hospital with complaints of fever, occipital headache, and vomiting.

Upon arrival, the patient was alert, with a Glasgow Coma Scale (GCS) score of 15 (E4V5M6), and no neurological abnormalities were noted. She continued to complain about occipital headaches. The vital signs were as follows: temperature, 38.6°C; blood pressure, 157/88 mmHg; heart rate, 91 bpm; respiratory rate, 24 breaths/min; and SpO₂, 94% on room air. Laboratory results on day 9 revealed an elevated white blood cell count (WBC: 14,720/μL) and a significantly elevated C-reactive protein (CRP: 18.56 mg/dL), implying ongoing inflammation. Procalcitonin levels were also elevated at 2.27 ng/mL, indicating a bacterial infection (Table 1).

**Table 1:** Blood Tests on Admission

Test	Result	Normal Range
White blood cell (WBC)	14,720/µL	4,000-10,000/µL
Hemoglobin (Hb)	14.1 g/dL	12-16 g/dL
Platelets (Plt)	165,000/µL	150,000-400,000/µL
C-reactive protein (CRP)	18.56 mg/dL	<0.3 mg/dL
Albumin (Alb)	3.0 g/dL	3.5-5.0 g/dL
Aspartate aminotransferase (AST)	65 IU/L	10-40 IU/L
Alanine aminotransferase (ALT)	54 IU/L	10-40 IU/L
Lactate dehydrogenase (LDH)	350 IU/L	140-280 IU/L
Alkaline phosphatase (ALP)	202 IU/L	44-147 IU/L
Total bilirubin (T-Bil)	1.3 mg/dL	0.1-1.2 mg/dL
Blood urea nitrogen (BUN)	18.6 mg/dL	7-20 mg/dL
Creatinine (Cre)	0.66 mg/dL	0.6-1.2 mg/dL
Estimated glomerular filtration rate (eGFR)	67.3 mL/min/1.73m^2^	>90 mL/min/1.73m^2^
Sodium (Na)	134 mEq/L	136-145 mEq/L
Potassium (K)	3.4 mEq/L	3.5-5.0 mEq/L
Chloride (Cl)	94 mEq/L	98-107 mEq/L
Calcium (Ca)	8.4 mg/dL	8.5-10.5 mg/dL
Phosphorus (P)	2.3 mg/dL	2.5-4.5 mg/dL
Glucose (Glu)	160 mg/dL	70-99 mg/dL
Hemoglobin A1c (HbA1c)	5.70%	4.0-5.6%
N-terminal prohormone of brain natriuretic peptide (NT-proBNP)	1751 pg/mL	<125 pg/mL
Procalcitonin	2.27 ng/mL	<0.05 ng/mL

Immunological markers and autoantibody tests were negative, except for a mildly positive lupus anticoagulant (LAC 1.3 (+)) (Table 2).

**Table 2:** Autoimmune-Related Tests

Test	Result	Normal Range
IgG	834 mg/dL	700-1,600 mg/dL
IgA	183 mg/dL	70-400 mg/dL
IgM	73 mg/dL	40-230 mg/dL
C3	147 mg/dL	90-180 mg/dL
C4	51 mg/dL	15-45 mg/dL
CH50	54.7 U/mL	30-45 U/mL
ANA	<40	Negative
RF	(-)	Negative
ACPA	(-)	Negative
MPO-ANCA	(-)	Negative
PR3-ANCA	(-)	Negative
LAC	1.3 (+)	Negative
CL-IgG	(-)	Negative
CL-IgM	(-)	Negative
β2-GP1	(-)	Negative

Urinalysis showed proteinuria (2+), hematuria (3+), and ketonuria (+), but no leukocytes or nitrites, ruling out urinary tract infection (Table 3).

**Table 3:** Urinalysis

Test	Result	Normal Range
Specific gravity	1.026	1.005-1.030
pH	5.5	4.5-8.0
Protein	2+	Negative
Occult blood	3+	Negative
Ketones	+	Negative
Glucose	±	Negative
Leukocytes	-	Negative
Nitrite	-	Negative

Cerebrospinal fluid analysis revealed a cell count of 8/3 μL, protein level of 36.0 mg/dL, and glucose level of 65 mg/dL, with no signs of infection.

**Figure 1:**
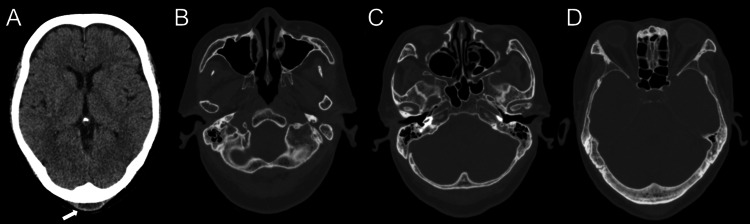
Initial Head CT A: A fluid collection is observed in the suboccipital region (arrow). No abnormal parenchymal absorption suggesting acute cerebrovascular diseases is seen. No space-occupying lesions in the skull. B, C, D: No abnormal findings in the sinuses, middle ear, mastoid air cells, and orbit. CT, computed tomography

A head computed tomography (CT) scan performed on day 9 revealed subcutaneous fluid collection in the occipital region, but no evidence of acute cerebrovascular diseases or parenchymal abnormalities. No mass lesions were noted within the skull, and no abnormalities were found in the paranasal sinuses, middle ear, mastoid, or orbits (Figure 1).

**Figure 2:**
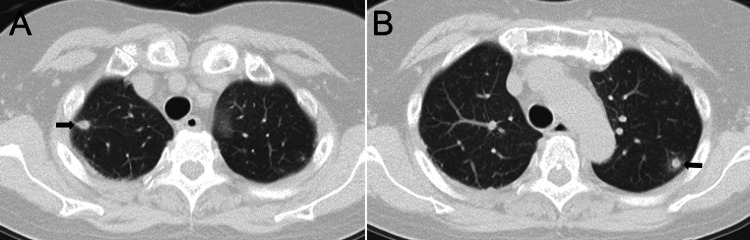
Chest CT A, B: Multiple nodules are observed in the peripheral lungs, with surrounding ground-glass opacities suggestive of a halo sign (arrow). CT, computed tomography

Chest, abdominal, and pelvic CT scans revealed multiple nodular opacities with halo signs in both lungs, suggesting an infectious or hematogenous process (Figure 2).

**Figure 3:**
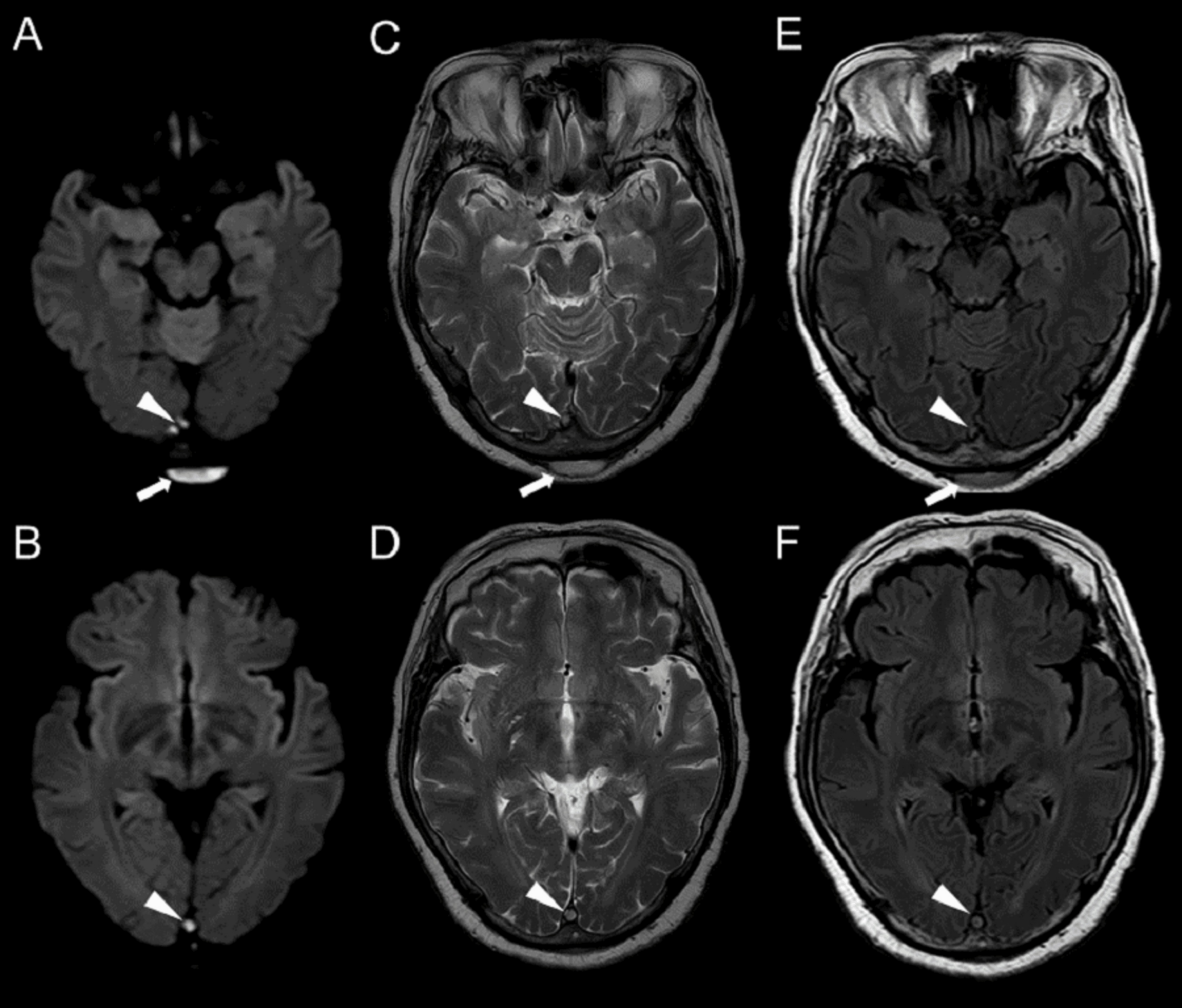
Head MRI A, B: DWI; C, D: T2WI; E, F: FLAIR A 2.4 cm flat fluid collection is observed in the suboccipital region (arrows). At the same level, a high signal is observed in the venous sinus on DWI. Findings are consistent with a suboccipital abscess and venous sinus thrombosis (arrow heads). MRI, magnetic resonance imaging; DWI, diffusion-weighted imaging; T2WI, T2-weighted imaging; FLAIR, fluid-attenuated inversion recovery

A head magnetic resonance imaging (MRI) on day 10 showed subcutaneous fluid collection with high signal intensity on diffusion-weighted imaging, confirming the presence of a subcutaneous abscess. Additionally, high signal areas were seen in the superior sagittal sinus and right sigmoid sinus, raising suspicion for venous sinus thrombosis (Figure 3).

**Figure 4:**
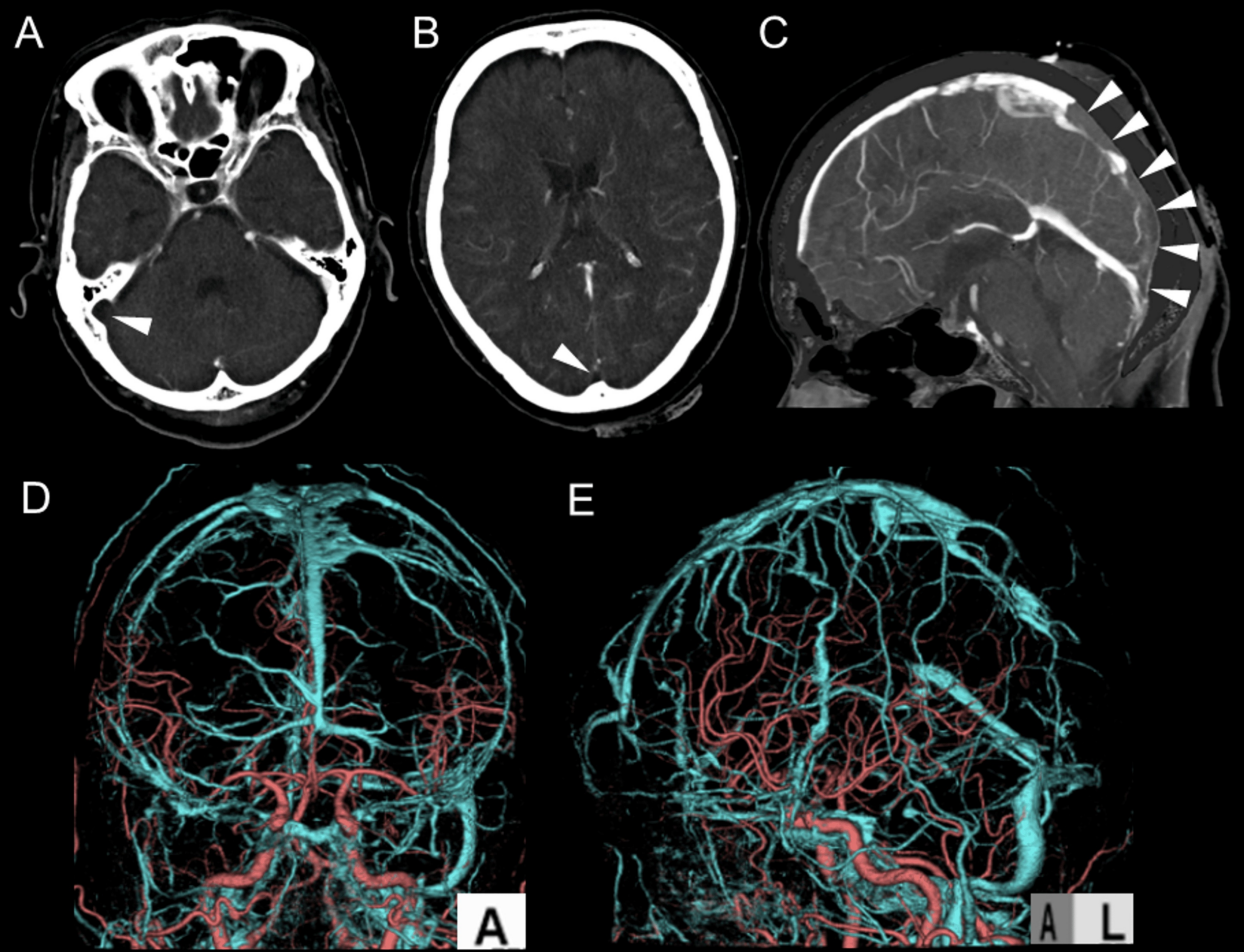
CTV (A, B, C), 3D CTA, and 3D CTV Fusion Images (D, E) Suspicion of thrombosis causing occlusion from the superior sagittal sinus to the right sigmoid sinus (arrow heads). CTV, computed tomography venography; CTA, computed tomography angiography

Computed tomography venography (CTV) confirmed the absence of contrast enhancement from the superior sagittal sinus to the right sigmoid sinus, establishing the diagnosis of venous sinus thrombosis (Figure 4).

A transthoracic echocardiogram showed no valvular vegetation or evidence of infective endocarditis. Although the LAC titer was mildly elevated, other antiphospholipid antibodies were negative. Given the clinical context, namely, MSSA bacteremia and subcutaneous abscess, the CVST was attributed to infectious thrombophlebitis rather than a hypercoagulable state. Thus, the mildly positive LAC was not the principal contributor to the thrombosis in this patient.

The patient was diagnosed with CVST secondary to MSSA bacteremia, originating from the occipital subcutaneous abscess. Empiric antibiotic therapy with ceftriaxone (2 g/day) was initiated on day 9. On day 10, incision and drainage of the occipital subcutaneous abscess were performed, and both blood and abscess cultures yielded MSSA. In response to the detection of gram-positive cocci in blood cultures, vancomycin (1 g twice daily) was added on the same day to ensure coverage for possible methicillin-resistant Staphylococcus aureus (MRSA). The patient also reported diplopia in the right eye on day 10. On day 13, based on susceptibility results confirming MSSA, the antibiotic regimen was de-escalated to cefazolin (2 g three times daily). However, due to persistent clinical symptoms and lack of radiological improvement in the CVST, the regimen was escalated to meropenem (2 g three times daily) on day 18 to broaden antimicrobial coverage. Following defervescence and a marked decrease in inflammatory markers, meropenem was continued for two weeks. On day 34, ceftriaxone (2 g twice daily) was reintroduced and administered for an additional two weeks to complete the antibiotic course.

**Figure 5:**
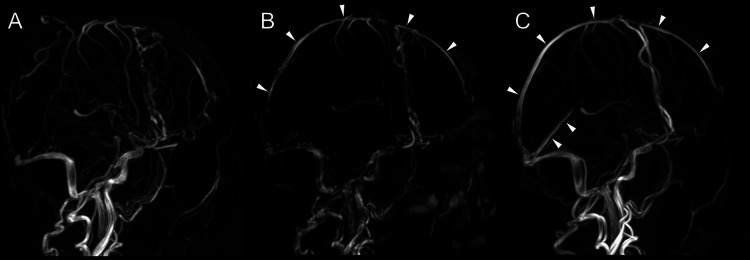
Temporal Changes in MRV A: One month after the event, MRV reveals filling defects in the superior sagittal sinus, straight sinus, right transverse sinus, and sigmoid sinus. B: At six months, recanalization of the superior sagittal sinus is observed (arrow heads). C: At 24 months, recanalization of both the superior sagittal sinus and straight sinus is noted (arrow heads). MRV, magnetic resonance venography

For the management of CVST, heparin was administered to achieve the target-activated partial thromboplastin time (APTT) range of two times the normal value, followed by warfarin, maintaining a PT-INR of 2 to 3. The patient’s headache improved, and she was discharged home on day 47. Thereafter, long-term anticoagulation therapy was continued. Six months later, the diplopia resolved, and magnetic resonance venography (MRV) revealed partial recanalization of the superior sagittal sinus. MRV performed 24 months after the onset showed complete recanalization of the superior sagittal sinus (Figure 5).

The patient is now transitioning to direct oral anticoagulants (DOACs) with gradual dose reduction.

## Discussion

In the present case, CVST extended from the suboccipital abscess to the adjacent dural venous sinuses, despite the absence of otitis media or sinusitis.

CVST is a condition in which a thrombus forms in the dural venous sinuses, the main venous outflow pathways of the brain, resulting in various neurological symptoms such as brain edema, venous infarction, intracranial hemorrhage, and seizures. Unlike arterial ischemic stroke, its pathophysiology is characterized by widespread edema and progressive dysfunction due to venous outflow obstruction [2-5].

CVST caused by infection (infective CVST) can also occur in elderly patients and often results in poor prognosis [2,6]. The most common causes of infective CVST are sinusitis and otitis media, with infection spreading to the cavernous or sigmoid sinus, as reported in previous cases [2-4]. Previous reports of CVST associated with subcutaneous abscesses have involved cases where infections originated from frontal sinusitis with spread to the frontal subgaleal space [7], or Bezold’s abscess extending from mastoiditis along the sternocleidomastoid muscle [8], all of which involved otolaryngologic infections. Only one previously reported case described infectious CVST arising from a subcutaneous abscess following repetitive head trauma without accompanying otitis media or sinusitis [9], making our case particularly rare.

Anatomically, the scalp and subcutaneous tissues are connected to the venous sinuses through emissary veins and diploic veins of the skull, allowing external infections to directly or hematogenously reach the venous sinuses [10]. In particular, the suboccipital region, which is connected to the sigmoid sinus and superior sagittal sinus via the occipital veins, has an anatomical basis for a suboccipital abscess to be the origin of venous sinus thrombosis. Furthermore, MSSA, the causative organism in this case, is commonly found in skin and soft tissue infections. It has a strong ability of toxin production and vascular invasion. MSSA's tissue-invasive properties increase under the conditions, for instance, immunocompromise or steroid use. Those conditions enhance the probability of bacteremia, abscess formation, and vascular infections. Direct invasion of the vascular endothelium, leading to thrombus formation, is thought to be a key factor in the pathophysiology of infective CVST [11]. In this case, steroid use may have suppressed the immune response, allowing the infection to spread through the bloodstream and reach the cerebral venous sinuses.

MRI with contrast and MRV are the first-line diagnostic modalities for CVST, and the use of DWI, T1-weighted imaging, and venous phase 3D CTA can enhance diagnostic accuracy [5]. In this case, we were able to diagnose early as DWI revealed hyperintensity within the venous sinus and CTV showed clear filling defects.

The primary treatment involves the selection and administration of appropriate antibiotics for the causative pathogen, along with surgical intervention to control the infection source [2-5]. In our case, skin incision and drainage were performed, and antibiotics were tailored to MSSA. Furthermore, even in infective CVST, anticoagulation therapy is generally recommended, and its combination with antibiotics can promote venous recanalization and reduce brain edema [3,5]. Our patient was transitioned from heparin to warfarin and then to a DOAC, with MRV confirming venous sinus recanalization 24 months after treatment. In the acute phase of CVST, unfractionated or low-molecular-weight heparin is the first-line anticoagulant due to its rapid onset of action and for ease of titration [12,13]. During the maintenance phase, warfarin is used to maintain an INR of 2.0-3.0 [12,13]. Guidelines recommend anticoagulation for three months in provoked CVST, six to 12 months in idiopathic or mild-risk cases, and indefinite therapy for recurrent or high-risk thrombosis [12]. Recently, DOACs have been increasingly used in CVST management, with studies supporting their efficacy and safety profiles [13-15].

Regarding neurological sequelae, temporary diplopia was observed in our patient; however, it resolved six months after the combined antibiotic and anticoagulation therapy, resulting in a favorable prognosis. Several studies stated that CVST can lead to residual symptoms such as optic nerve compression, cranial nerve dysfunction, and seizures; therefore, long-term neurological follow-up is recommended [5,16].

This case highly indicates the possibility of rare clinical course, which a localized infection rapidly spreads to the cerebral venous sinuses via the venous system. In cases of persistent headache and/or fever with a background of infection, even if the infection is limited to a subcutaneous abscess, it is crucial to take CVST into consideration and perform timely imaging studies. Additionally, proper anticoagulation therapy and infection source control can lead to complete recovery without sequelae, as demonstrated in this case.

## Conclusions

This case represents a rare complication of extensive CVST, extending from the superior sagittal sinus to the right sigmoid sinus, triggered by MSSA bacteremia originating from an occipital subcutaneous abscess without any associated otolaryngologic infection. Although the infection was initially localized to the skin and subcutaneous tissue, this case highlights that the anatomical continuity and the invasiveness of the pathogen can facilitate the spread of the infection to intracranial structures. It underscores the importance of considering CVST in patients with persistent fever and/or headache, even when the primary infection appears confined to superficial soft tissues.
